# The Effects of Temperature and Time of Heat Treatment on Thermo-Mechanical Properties of Custom-Made NiTi Orthodontic Closed Coil Springs

**DOI:** 10.3390/ma15093121

**Published:** 2022-04-26

**Authors:** Thanate Assawakawintip, Peerapong Santiwong, Anak Khantachawana, Kawin Sipiyaruk, Rochaya Chintavalakorn

**Affiliations:** 1 Department of Orthodontics, Faculty of Dentistry, Mahidol University, Bangkok 10400, Thailand; thanate.assawakawintip@gmail.com (T.A.); peerapong.san@mahidol.ac.th (P.S.); kawin.sip@mahidol.ac.th (K.S.); 2 Department of Mechanical Engineering, Faculty of Engineering, King Mongkut’s University of Technology, Bangkok 10140, Thailand; anak.kha@kmutt.ac.th

**Keywords:** closed coil springs, nickel-titanium, orthodontics, superelasticity, thermo-mechanical properties

## Abstract

Nickel-Titanium (NiTi) springs have been increasingly used in orthodontics; however, no optimum condition of heat treatment has been reported. Therefore, this research was conducted to determine the optimum heat-treatment temperature and duration for the fabrication of NiTi-closed coil springs by investigating their effects on thermo-mechanical properties. As-drawn straight NiTi wires of 0.2 mm diameter were used to fabricate closed coil springs of 0.9 mm lumen diameter. The springs were heat-treated at three different temperatures (400, 450, and 500 °C) with three different durations (20, 40, and 60 min). Electron Probe Micro-Analysis (EPMA) and Differential Scanning Calorimetry (DSC) were used to investigate element composition and thermo-mechanical properties, respectively. Custom-made NiTi closed coil springs composed of 49.41%-Ti and 50.57%-Ni by atomic weight, where their DSC curves of 500 °C presented the obvious endothermic and exothermic peaks, and the austenite finish temperature (A_f_) were approximately 25 °C. With increasing temperature, deactivation curves presented decreased plateau slopes generating higher superelastic ratios (SE ratios). At 500 °C, closed coil springs showed superelastic tendency with lower stress hysteresis. The thermo-mechanical properties were significantly influenced by heat-treatment temperature rather than duration. The optimum parameter appeared to be 500 °C for 40 min to produce appropriate force delivery levels, relatively low plateau slope, and lower hysteresis for orthodontic use.

## 1. Introduction

Nickel-Titanium (NiTi) alloy has been increasingly used in orthodontic practice, including archwires, brackets, and coil springs. NiTi can be considered as one of the main materials in orthodontics as it delivers low and constant force over long activations [1,2], resulting from its two unique properties: superelasticity and shape memory effects [3]. With light and continuous force, NiTi appears to be efficient for tooth movement with minimal irreversible damage to root structure, periodontal ligament, and alveolar bone [3]. In addition, NiTi appears to have superior properties over elastomeric chains and closing loop mechanics in terms of less force decay and it is clinically less time consuming [4,5]. Therefore, NiTi can be used as closed coil springs for a space closure technique in orthodontic practice. As NiTi closed coil springs seem to be limited for selection, more available options should be considered to provide an optimum force for varied orthodontic movement conditions.

There appears to be a number of thermo-mechanical properties of NiTi closed coil springs as requirements for orthodontic practice. Constant force delivery with good superelastic ratio (SE ratio) and low hysteresis should be required [6]. Moreover, A_f_ should be slightly lower than oral temperature (37 °C) in order to present superelasticity in oral environments [7,8]. These mechanical properties of NiTi closed coil springs can be illustrated in a load-extension curve, which is relevant to austenitic and martensitic transformation [9]. Austenite is stable in high temperature and low stress, while martensite is more stable in low temperature and high stress [10]. These two structures can be reversed back and forth called ‘phase transformation’, which could be induced by either temperature or stress.

The phase-transformation temperatures are defined as Martensite start (M_s_), Martensite finish (M_f_), Austenite start (A_s_), and Austenite finish (A_f_) and can be investigated by differential scanning calorimetry (DSC) [11]. Above A_f_ temperatures, NiTi coil springs will be in austenitic phase, which exhibit superelasticity that allow the springs to stretch in longer distance without distortion [11,12]. Being stretched, if the spring extension increases beyond the elastic point, there will be the elastic distortion. However, the material can still return to its original shape (so called ‘pseudoelasticity’) [13], where phase transformation occurs as ‘stress-induced martensite’. On the other hand, if spring extension rises over the elastic limit, there will be permanent deformation, and the material will then break down when it proceeds beyond the failure point.

In addition to nickel composition in the alloy, wire diameter, and coil spring diameter, the thermo-mechanical properties of NiTi can be influenced by heat-treatment temperature and duration [2,14]. Longer duration and higher temperatures tend to anneal the product but result in more exact shapes with less springback [15]. With the long extension of pseudoelasticity of NiTi, the 300–500% extension of NiTi-closed coil springs (approximately 12–15 mm) has been reported to not reach the permanent deformation and can return to their original shape in clinical orthodontic situations [2,16]. Thus, the balance of heat-treatment temperature and the duration of the shape setting process should be concerned to provide appropriate mechanical properties of springs, including stress release and shape memory.

Although the conditions of approximately 350–550 °C for up to 60 min have been speculated for heat treatment after coiling for coil shape preservation [17], there was no report of the optimum condition of heat treatment due to commercial secrets. As a result, to customize coil springs, it is necessary to investigate the optimum heat treatment condition with low temperature and short period while being sufficient to produce good endurance and superelasticity. While the literature in the development procedure of this orthodontic material is still limited [18], this research was conducted to investigate the effects of temperature and time of heat treatment on the thermo-mechanical properties of custom-made NiTi closed coil springs according to orthodontic requirements. The knowledge and understanding retrieved from this research would eventually provide the most clinically effective NiTi coil springs.

## 2. Materials and Methods

### 2.1. Fabrication of NiTi Closed Coil Springs

**Figure 1:**
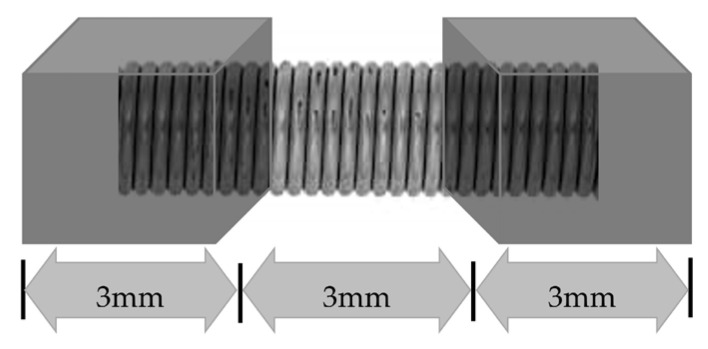
A custom-made NiTi closed coil spring with a middle 3 mm span of active length attached with acrylic blocks on both ends.

NiTi closed coil springs were custom-made by using a larger dimension as-drawn straight wires from Furukawa Techno Material Co. Ltd. (Hiratsuka, Japan) and were cold drawn to 0.2 mm diameter. An as-drawn wire, which initially was in austenite, was wound and fixed around a pin, fabricating coil springs with 0.9 mm-diameter lumen. They were then heat-treated to retain the shape with nine conditions by three different temperature settings (400, 450, and 500 °C), with three different times (20, 40, and 60 min). After heat treatment, all springs were then quenched in ice-cold water. The closed coil springs were cut into 9 mm length pieces and 3 mm in the middle part was left as an active part; the remaining 3 mm parts on both ends were fixed into acrylic blocks (Figure 1).

### 2.2. Electron Probe Micro-Analysis (EPMA)

To determine chemical composition, ten pieces of an as-drawn NiTi wire and a commercial NiTi closed coil spring were tested by Electron Probe Micro-analysis or EPMA (JEOL Model JXA-8100, Tokyo, Japan) with the following machine conditions: accelerating Voltage 15 kV, current 2.50 × 10^−8^ A, and probe diameter 0–1 μm. To quantitatively analyze target elements, the correction method was ZAF (Z = atomic number correction, A = Absorption correction, and F = characteristic fluorescence correction).

### 2.3. Differential Scanning Calorimeter (DSC)

One specimen of each condition approximately 10–20 mg was submitted to investigate the transformation temperature range (TTR) by a method of differential scanning calorimeter (Mettler Toledo Stare System DSC 2 Module, Greifensee, Switzerland) with a temperature range from −80 °C to 100 °C at a rate of 10 °C/min. Martensitic, rhombohedral, and austenitic phases (M_f_, M_s_, R_Mf_, R_Ms_, R_As_, R_Af_, A_s_, and A_f_) were observed by the construction of the intersection between the baseline and the tangent of each peak.

### 2.4. Tensile Tests

**Figure 2:**
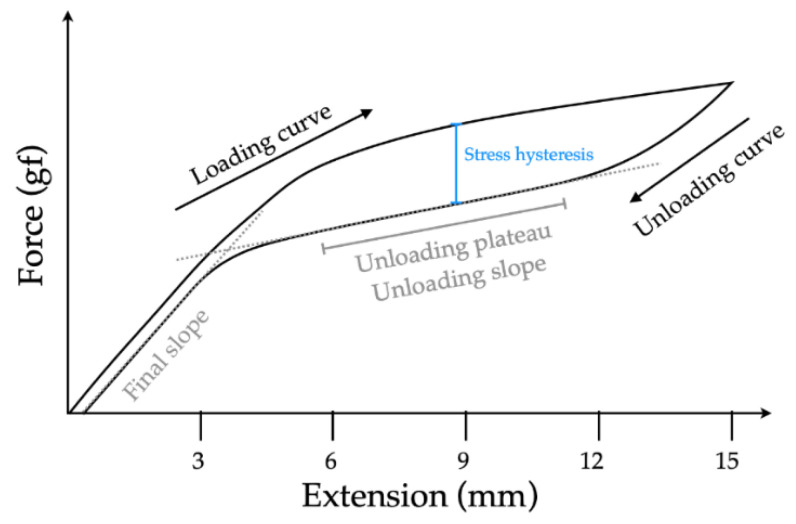
A load-extension curve illustrating loading and unloading curve, unloading plateau/slope, and final slope of deactivation curve, as well as stress hysteresis.

For thermo-mechanical testing, five specimens of each condition and five commercially available NiTi closed coil springs (Extra light, 0.23 mm wire diameter and 0.9 mm spring lumen, TOMY International Inc., Tokyo, Japan, as a control) were tested with a universal testing machine (Mecmesin’s MultiTest 2.5-I, 25 N of load cell) in a temperature-controlled water bath (37 ± 1 °C). To minimize the experimental errors from a mechanical role of the fixture, the specimens were pre-stretched to a force level of 0.2 N. The springs afterwards were stretched from the initial active length of 3 mm to 18 mm (500% of activation length) with a crosshead speed of 10 mm/minute and then reversed to the original length. Load-extension curves both activation and deactivation were plotted at 0.5 mm interval (Figure 2).

### 2.5. Data Analysis

The force exertions at deactivation lengths of 3, 6, 9, and 12 mm were collected. The means and standard deviations of the force exertion were also calculated for each deactivation length. The statistical analysis of force delivery on deactivation curves at each deactivation length were performed by the Kruskal–Wallis test and Dunn’s pairwise comparisons. The statistical significance was taken at *p*-value < 0.05. A plateau phase was determined by the most horizontal segment of a deactivation curve using the best-fitted straight line with superimposition, which was observed by a linear regression model (Microsoft Corporation, Redmond, WA, USA. Microsoft Excel for Mac. 2019), and the SE ratio was calculated by the formula: the final slope of the deactivation curve divided by the plateau slope [6]. The stress hysteresis was calculated by subtracting the deactivation force from activation force at the activation length of 6, 9, and 12 mm.

## 3. Results

### 3.1. Composition of NiTi Materials

The compositions of commercial and custom-made NiTi closed coil spring investigated by EPMA (JEOL Model JXA-8100, Tokyo, Japan) methods are presented in Table 1. Custom-made NiTi springs composed of 49.41 ± 0.21%-Ti and 50.57 ± 0.22%-Ni, while commercial NiTi coil spring comprised 48.33 ± 0.27%-Ti and 51.64 ± 0.27%-Ni by atomic weight.

### 3.2. Transformation Temperature Range (TTR)

**Figure 3:**
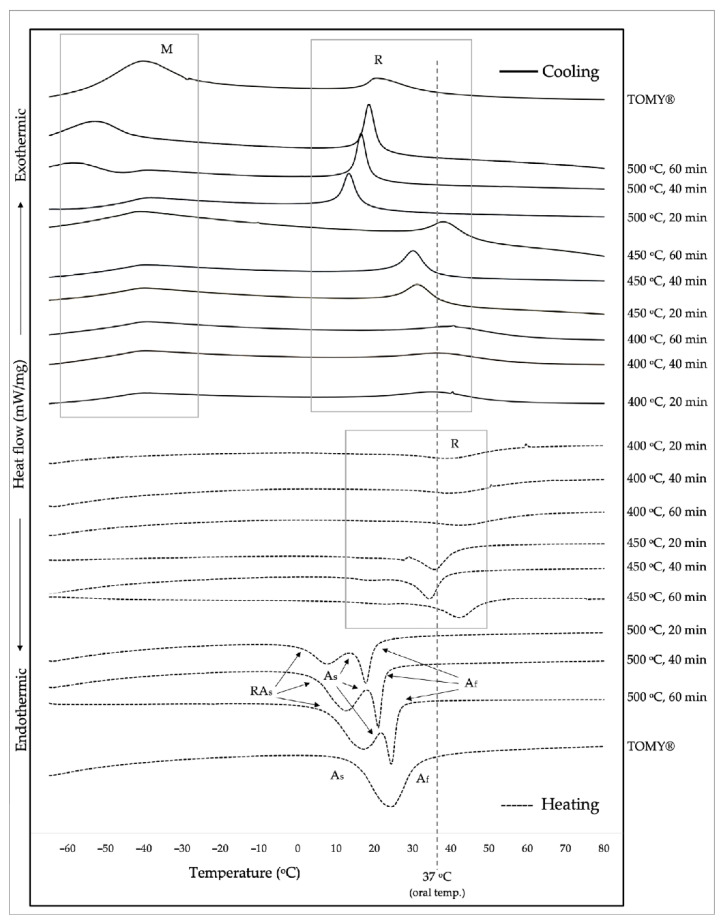
Cooling (as solid lines) and heating (as dash lines) curves of custom-made and commercial NiTi closed coil springs under different temperatures and times of heat treatment (comparable with oral temperature 37 °C); M = Martensitic phase, R = Rhombohedral phase, A_s_ = Austenite start temperature, A_f_ = Austenite finish temperature, RA_s_ = Rhombohedral reverse start temperature.

All heat treatment conditions of custom-made and commercial NiTi closed coil springs were shown in Figure 3, presenting the cooling phase as solid lines and the heating phase as dash lines. They were also used to evaluate TTR (M_f_, M_s_, R_Mf_, R_Ms_, R_As_, R_Af_, A_s_, and A_f_), as demonstrated in Table 2. On the cooling phase, the exothermic peaks of R-phase and M-phase were more evident with increasing temperatures of heat treatment from 400 °C to 500 °C, and TTR appeared to decrease with increasing temperature. On the heating phase, DSC curves could be categorized into three groups depending on heat-treatment temperatures. The R-phase of heat treatment at 400 °C showed broad and low endothermic peaks, while at 450 °C, it presented higher peaks. The endothermic peaks of 500 °C were observed more clearly and presented as two peaks by R-phase and A-phase. The A_f_ of 400 °C and 450 °C was unable to identify heat-treatment temperatures up to 80 °C, whereas the A_f_ of 500 °C was observed at around 20 °C to 26 °C. The TOMY^®^ spring (Tokyo, Japan) presented only one endothermic peak, and A_f_ was around 30 °C. The results demonstrated that the A-phase could be obviously found in the springs heat-treated at 500 °C, and TTR decreased when increasing heat-treatment temperatures but was less influenced by time.

### 3.3. Mechanical Properties: Force Delivery, Plateau Slope, SE Ratio, and Stress Hysteresis

#### 3.3.1. Force Delivery

**Figure 4:**
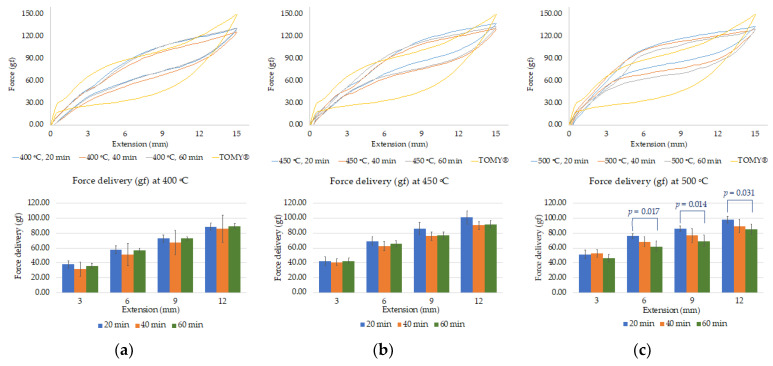
Superimpose of load-extension curves of custom-made closed coil springs under different heat treatment conditions with commercial NiTi closed coil springs (TOMY^®^): (**a**) at a constant temperature (400 °C) with various durations; (**b**) at a constant temperature (450 °C) with various durations; (**c**) temperature (500 °C) with various durations.

**Figure 5:**
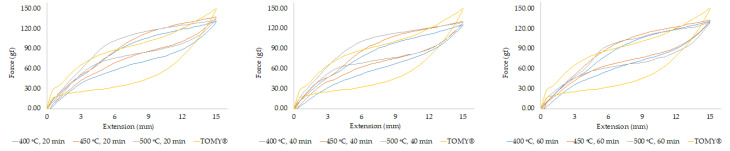

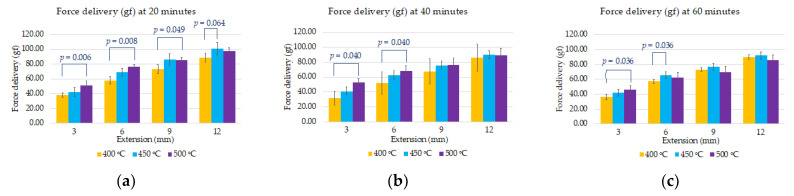
Superimpose of load-extension curves of custom-made NiTi closed coil springs under different heat treatment conditions with commercial NiTi closed coil springs (TOMY^®^); (**a**) at a constant time (20 min) with various temperatures; (**b**) at a constant time (40 min) with various temperatures; (**c**) at a constant time (60 min) with various temperatures.

The load-extension curves were generated, presenting a typical curve of superelastic materials showing the plateau force level on both activation and deactivation curves (Figure 4 and Figure 5). The force exertion of unloading curves was observed at the deactivation lengths of 3, 6, 9, and 12 mm. The result demonstrated that force delivery of each condition ranged from 31 g to 101 g, compared to commercial closed coil springs (TOMY^®^) exerting 25 g to 79 g of force.

There were no significant differences in force delivery between heat treatment at 400 °C and 450 °C (*p* > 0.05) at any durations as shown in Figure 4a,b, while Figure 4c illustrated significant differences between heat treatment at 500 °C/20 min and 500 °C/60 min, as the force significantly decreased following an increase in heat-treatment time (*p* < 0.05) of about 12–16 g. These results demonstrated that the effect of heat-treatment time on force delivery can be found only at 500 °C.

When considering a constant treatment time to determine the effect of heat-treatment temperature, Figure 5A shows statistically significant increases of force delivery when increase heat-treatment temperature from 400 °C to 500 °C, especially in 20 min of heat treatment at almost extension of springs. According to these results, an increase in heat-treatment temperature tended to improve force delivery, whereas time seemed to have a smaller effect.

#### 3.3.2. Plateau Slope, SE Ratio, and Stress Hysteresis

A plateau phase of deactivation curves was determined by the most horizontal segment of a deactivation curve using the best-fitted straight line superimposed on it. The results represented that the condition of 500 °C/40 min generated the lowest plateau slope among custom-made NiTi closed coil springs as of 2.88 and the highest value of SE ratio as of 8.06, representing ‘Superelasticity’. In other conditions, the ratios indicated that springs provided ‘Superelastic tendency’ (SE ratio = 2.09–6.61). At 500 °C of heat treatment, the greater value of SE ratio as of 5.63–8.06 was found; the higher the temperature of heat treatment, the higher the reported SE ratio. Compared to commercial closed coil springs, TOMY^®^ springs provided the least plateau slope as of 2.76 with highest SE ratio as of 13.79 indicating ‘Superelasticity’, as shown in Table 3.

Stress hysteresis calculated at 6, 9, and 12 mm of activation length showed that TOMY^®^ springs presented the highest value ranging from 41 g to 55 g, while custom-made springs showed significantly lower hysteresis ranging from 17 g to 38 g.

## 4. Discussion

Nickel–titanium appears to be the most popular shape memory alloy (SMAs) in the biomedical field, which can exhibit unique properties that has drawn the interest of various scientific communities and industries [19]. An understanding of the thermo-mechanical properties of the material is required to modify NiTi alloys for clinical use. The custom-made NiTi coil springs in this study consisted of 50.57%-Ni compared to 51.64%-Ni in commercially available springs (TOMY^®^). Previous studies have shown that alloy composition, wire diameter, lumen size, and martensitic transformation temperature affect the thermo-mechanical properties of NiTi alloys [2,15]. The processing of NiTi alloys, such as hot-working, cold-working, annealing, and aging, is also a contributing factor. For instance, impurities that result from the melting process can influence the Ni:Ti ratio, where an increase in Ni proportion would tend to reduce TTR [15]. In addition, a slight change in chemical composition will affect thermomechanical properties [15]. Although higher TTR would be expected in our custom-made springs due to the lower Ni component, we found that the A_f_ of our springs was not significantly higher than the TOMY^®^ springs. These findings might have been resulted from the stress from cold working and heat treatment processes carried out for our NiTi springs.

DSC curves of our custom-made springs, which were heat treated at different temperatures (400, 450, and 500 °C) and durations (20, 40, and 60 min), were compared to commercial springs (TOMY^®^) and illustrated in Figure 3. According to the DSC curves, the NiTi wires used in this study were processed by cold drawing, creating remaining internal residual stresses existing in an intermediate phase known as the ‘Rhombohedral phase or R-phase’. The residual stress from cold working can be reduced by heat treatment, and the R-phase’s peak temperature would be increased from an increase in internal residual stress [20]. In other words, increasing heat treatment procedures either by increasing temperature or time can reduce internal residual stress and causes a decrease in R-phase peak temperature.

With heat treatment at low temperatures such as 400 °C, the observed peak found in our research relatively demonstrated low temperature hysteresis. In addition, an increasing duration from 20 to 60 min could result in changes of the peak temperatures and amplitudes, informing that the heat treatment at 400 °C could not significantly relieve residual stress. As the residual stress could interfere and impede the phase transformation of material, these springs would have higher strength than those with heat treatment at higher temperatures. The stress-induced martensite would also be increased. At higher treatment temperatures such as 450 °C, which is lower than recrystallization temperature (526 °C) [21], DSC curves were clearly observed with R-phase transformation. When increasing time from 20 to 60 min, the TTR of R-phase seemed to be increased. Although M-phase transformation cannot be observed, even the temperature has been reduced to −60 °C, and broad peaks can be seen at about −40 °C. According to these results, heat treatment at 450 °C could relieve some internal residual stresses greater than heat treatment at 400 °C, resulting in a higher chance to have phase transformation.

The DSC data of the 500 °C heat treatment condition in this study, which is close to the recrystallization temperature (526 °C), demonstrated that the residual stress from cold drawing process was reduced more significantly than other groups, resulting in an easier occurrence of martensite transformation. As recrystallization appears to be retrieved when annealed at 500 °C, grain growth can cause the improvement of hardness and superelasticity following increasing annealing temperatures [22]. The peak of martensitic phase was clearly visible even though the amplitudes were very low. During the process of healing the specimens, two peaks of phase transformation as rhombohedral and austenite were very close. With increasing time, A-phase peaks increased and appeared to be more overlapped with R-phase peaks, rendering the reverse peak of rhombohedral invisible. This indicates that, with an increased reduction in residual stress due to an increase in treatment temperature and duration, a higher chance for phase transformation to occur exists. Compared to commercial springs (TOMY^®^), the DSC curves of our custom-made coil springs with the condition of 500 °C/60 min showed relatively similar results.

The heat treatment for cold-worked metals can reduce residual stresses and, consequently, flattens the plateau slopes, resulting in more constant force delivery, which can be considered as appropriate for orthodontic tooth movement. The behavior of the alloy can be influenced by cold working prior to heat treatment, where a higher level of cold working produces more strain hardening and ultimate tensile strength [23]. According to our DSC data, heat treatment at 500 °C could relieve more residual stress than the temperatures of 400 °C and 450 °C, where there was a high chance to have a phase transformation resulting in a lower slope of deactivation curve. Thus, our 500 °C heat-treated NiTi coil springs could produce a higher SE ratio with lower A_f_.

Our findings reported a slight increase in the strength of NiTi wires with the heat treatment at lower temperatures (410–435 °C), while there was a reduction in strength at higher temperatures (485–540 °C). These findings are consistent with the previous literature [24]. There can be wire strengthening due to a higher Ti_11_Ni_14_ precipitate nucleation rate with heat treatment at lower temperatures. These precipitates act as barriers to dislocation motions and, thus, strengthen the alloy matrix. On the other hand, the precipitation rate decreases at a higher temperature range. Higher temperatures at around 475 °C can also promote the onset of recrystallization, and Ti_11_Ni_14_ precipitates are replaced by Ti_3_Ni_2_ plates, which are less effective in strengthening the alloy [15,25]. Therefore, lower heat-treatment temperatures tend to produce superior shape memory characteristics.

Apart from the unloading force, the SE ratio is another parameter used to quantify the superelasticity of NiTi coil springs. In our study, the ending slopes of deactivation curves were used to calculate the SE ratio as they appeared to be more predictable and reliable. Moreover, the ending slopes were more parallel and corresponded to the initial stage of the activation curves, suggesting better precision. The SE ratio in this study was calculated by dividing the ending slope by the plateau slope of deactivation curve. The highest SE ratio of 5 to 8 was found from the heat-treatment condition of 500 °C for our custom-made NiTi coils springs. Based on the classification proposed by Segner et al. [6], these SE ratios can be interpreted as showing superelastic tendencies and superelasticity. However, the higher SE ratio of TOMY^®^ (13.79, indicated as superelasticity) was mainly caused by the steep ending slope, whereas the plateau slope was relatively similar to our custom-made NiTi coil springs treated at 500 °C. As a result, both TOMY^®^ and 500 °C heat-treated NiTi coil springs could deliver constant forces over a distance of 6 to 12 mm which are suitable to be used for canine retraction in actual clinical use.

Stress hysteresis in this research was the difference of the loading and unloading forces at any point of extension. Low stress hysteresis is ideal for orthodontic tooth movement, as more reproducible clinical results can be achieved, chairside time can be reduced, and anchorage can be better conserved [26]. Hysteresis can be modified by the addition of a ternary element, such as iron and copper to lower it or niobium to widen it [27,28]. The use of higher heat-treatment temperatures over 300 °C to 500 °C has shown to have higher stress hysteresis due to the increasing grain size [29]. Although the pattern of change in stress hysteresis by increasing the heat-treatment temperature from 400 °C to 500 °C in our study was not consistent, a longer heat-treatment times from 20 to 60 min at 450 °C and 500 °C resulted in a gradual increase in stress hysteresis.

The heat-treatment temperatures of 500 °C used in this research demonstrated the most suitable A_f_ of 20–26 °C for clinical use in orthodontic practice. As orthodontic applications, the A_f_ of NiTi alloys should be slightly lower than oral temperature to allow the material to exhibit superelastic behavior. Ideal force magnitudes for the retraction of canine teeth were previously suggested to be between 150 g and 260 g [30,31,32], but more recent evidence suggests that force levels as low as 50 g to 100 g were sufficient [33,34]. Regardless of the duration of heat treatment at 500 °C, our custom-made NiTi springs exerted approximately 51–98 g of force over a deactivation distance between 3 mm to 12 mm, which is suitable for tooth movement and is a relatively constant force over long activations. Although stress hysteresis increased with more prolonged heat treatment at 500 °C, no significant changes were reported either in the slope of the plateau or the SE ratio. Therefore, according to the NiTi coil springs fabricated in our study, heat treatment at 500 °C with 40 min would produce the most ideal thermo-mechanical properties with low and constant forces (51–98 g), the lowest plateau slope (2.88) with the highest SE ratio (8.06), low stress hysteresis, and suitable A_f_ (31.83 °C) for applications in orthodontic tooth movement. All of these parameters, representing clinical requirements, were investigated in this research to obtain the optimum condition for custom-made NiTi coil springs.

Despite the strengths of our research, Ni compositions of our custom-made and commercial NiTi coil springs (TOMY^®^) were considerably different. As TOMY^®^ springs had been investigated in this study due to its wide use in orthodontics, further research may select a similar chemical composition of materials. Research in microstructure should also be conducted to investigate the amount of Ni in solid solution and precipitation in order to provide detailed information of the thermo-mechanical properties. In addition, this in vitro study had a limitation in simulating human oral environment and behaviors. As corrosion properties of NiTi alloys appeared to be affected by the pH of saliva and fluorides [35], further research in clinical settings should be required to evaluate our custom-made springs to ensure its effectiveness in orthodontic practice.

## 5. Conclusions

The thermo-mechanical properties of custom-made NiTi closed coil springs can be significantly influenced by the temperature of heat treatment rather than by duration. The appropriate heat treatment conditions for fabricating Ti-50.57%Ni closed coil springs should be 500 °C for 40 min in order to produce the optimum level of force delivery, relatively low plateau slope, lower hysteresis, and proper A_f_ for orthodontic use. The different chemical compositions between TOMY^®^ coil spring and as-received NiTi can lead to the difference in the TTR of NiTi alloys. Moreover, to memorize the coil shape, the spring is heat treated, which will release stress and sometimes recrystallization depending on heat treatment conditions. Even if there are differences in chemical composition and DSC curve, the load-extension curve of custom-made NiTi closed coil springs can exhibit favorable characteristics including superelastic and shape memory effects after heat treatment at 500 °C. Finally, the lower stress hysteresis of custom-made NiTi coil spring can be considered as beneficial for clinical use in orthodontic treatment, as there appears to be less negative impact of fluctuating temperature on NiTi force in an oral environment.

## Figures and Tables

**Table 1:** Composition of custom-made and commercial NiTi closed coil springs.

Sample	Percentage by Atomic Weight	
	Ni	Ti
Custom-made NiTi	50.57 ± 0.22	49.41 ± 0.21
TOMY^®^	51.64 ± 0.27	48.33 ± 0.27

**Table 2:** The Transformation Temperature Range (TTR) of custom-made and commercial NiTi closed coil springs (TOMY^®^).

Heat Treatment Condition (°C, min)	M_f_ (°C)	M_s_ (°C)	R_Mf_ (°C)	R_Ms_ (°C)	R_As_ (°C)	R_Af_ (°C)	A_s_ (°C)	A_f_ (°C)
400, 20	−56.17	−16.50	19.83	53.50	29.70	54.00	-	-
400, 40	−57.33	−13.33	24.33	58.50	29.33	59.16	-	-
400, 60	−54.00	−14.00	30.00	59.16	32.50	60.67	-	-
450, 20	−52.50	−13.50	25.00	39.16	30.83	43.33	-	-
450, 40	−54.83	−13.50	25.00	35.83	29.33	38.67	-	-
450, 60	−56.5	−0.66	32.5	47.00	36.33	49.33	-	-
500, 20	−53.17	−13.00	10.00	17.17	0.00	12.33	15.50	20.33
500, 40	-	−47.67	14.00	19.17	4.00	17.17	19.17	23.00
500, 60	-	−42.17	16.00	21.67	7.83	21.17	22.67	26.33
TOMY^®^	−56.17	−25.67	16.67	37.33	-	-	14.83	31.33

**Table 3:** Plateau slope, SE ratio, and means of stress hysteresis at different heat treatment conditions.

Conditions (°C, min)	Plateau Slope	SE Ratio	Stress Hysteresis (gf ± S.D.)		
			6 mm	9 mm	12 mm
400, 20	5.11	2.76	26.77 ± 4.25	33.59 ± 4.00	30.49 ± 3.35
400, 40	5.54	2.09	26.19 ± 7.01	31.37 ± 4.25	25.28 ± 5.00
400, 60	5.67	2.50	24.93 ± 3.74	33.97 ± 3.67	30.02 ± 2.81
450, 20	5.10	3.06	17.10 ± 2.22	28.50 ± 2.03	26.83 ± 0.58
450, 40	4.21	3.87	23.71 ± 2.98	33.74 ± 2.98	30.32 ± 2.91
450, 60	4.27	3.63	25.46 ± 3.43	35.42 ± 2.77	31.01 ± 1.19
500, 20	3.44	5.63	26.15 ± 3.43	31.31 ± 2.31	27.74 ± 3.38
500, 40	2.88	8.06	32.68 ± 3.57	36.82 ± 4.89	31.83 ± 3.67
500, 60	3.08	6.61	32.91 ± 7.96	38.61 ± 7.34	33.44 ± 4.25
TOMY^®^	2.76	13.79	55.12 ± 2.55	55.47 ± 2.68	41.65 ± 3.33

## Data Availability

The data are available upon request from the corresponding author.
